# MiR-93 suppresses tumorigenesis and enhances chemosensitivity of breast cancer via dual targeting E2F1 and CCND1

**DOI:** 10.1038/s41419-020-02855-6

**Published:** 2020-08-14

**Authors:** Chang Bao, Jishun Chen, Danni Chen, Yunkun Lu, Weiyang Lou, Bisha Ding, Liang Xu, Weimin Fan

**Affiliations:** 1 Program of Innovative Cancer Therapeutics, Division of Hepatobiliary and Pancreatic Surgery, Department of Surgery, First Affiliated Hospital, College of Medicine, Zhejiang University, Hangzhou, 310003 China; 2 Key Laboratory of Organ Transplantation, Hangzhou, 310003 China; 3 Key Laboratory of Combined Multi-organ Transplantation, Ministry of Public Health, Hangzhou, 310003 China; 4 Department of Cell Biology and Program in Molecular Cell Biology, College of Medicine, Zhejiang University, Hangzhou, 310000 China; 5 Department of Radiation Oncology, First Affiliated Hospital, College of Medicine, Zhejiang University, Hangzhou, 310003 China; 6 Life Sciences Institute, Zhejiang University, Hangzhou, 310000 China; 7 Clinical Research Center, First Affiliated Hospital of Zhejiang University College of Medicine, Hangzhou, 310003 China; 8 Department of Pathology and Laboratory Medicine, Medical University of South Carolina, Charleston, SC 29425 USA

**Keywords:** Breast cancer, Breast cancer, Apoptosis, Apoptosis

## Abstract

Chemoresistance of tumors often leads to treatment failure in clinical practice, which underscores pivotal needs to uncover novel therapeutic strategies. Accumulating evidences show that microRNAs (miRNAs) are widely involved in carcinogenesis, but their function on chemoresistance remains largely unexplored. In this study, we found that miR-93-5p (miR-93) significantly inhibited cell proliferation, induced G1/S cell cycle arrest and increased chemosensitivity to paclitaxel (PTX) in vitro and in vivo. Moreover, two well-established oncogenes, E2F1 and CCND1, were identified as dual targets of miR-93. Knockdown of E2F1 and CCND1 reduced cell proliferation and PTX-sensitivity, whereas overexpression of them had the opposite effect. More importantly, overexpression of E2F1 and CCND1 antagonized miR-93-mediated cell cycle arrest and apoptosis. Further mechanistic study revealed that miR-93 exhibited its inhibitory role by directly targeting E2F1 and CCND1 to inactivate pRB/E2F1 pathway and AKT phosphorylation. Taken together, our findings suggested that miR-93 greatly improved chemosensitivity and potentially served as a novel therapeutic target for breast cancer treatment.

## Introduction

Breast cancer is the leading cause of morbidity and mortality among female malignancies, remaining a worldwide public health concern^1^. Treatments of breast cancer include tumor resection, radiation and chemotherapy, alone or in combination usage. Among these therapies, chemotherapy is widely used for clinical application, including endocrine, cytotoxic, and targeted drugs^2^. However, most of patients succumb to chemoresistance, resulting in treatment failure and disease relapse. In clinical practice, acquired chemoresistance is more frequent, which means tumors are initially sensitive to chemotherapy, but after several courses, gradually become resistant to one or multiple drugs^3^. Therefore, it is desirable to better understand the genetic alterations and molecular mechanisms of chemoresistance, and to develop novel strategies to improve the therapeutic effect of anticancer drugs.

MiRNAs are a group of non-coding, single-stranded RNAs, with 19–25 nucleotides (~22 nt) in length. The mature miRNAs post-transcriptionally suppress the expression of target genes by directly binding to the 3′-untranslated regions (3′-UTRs) of target messenger RNAs (mRNAs), leading to translational blockade or mRNA degradation^4^. Numerous miRNAs function as gene regulators involved in multiple biological processes, such as cell proliferation, apoptosis, migration, and invasion^5^. Furthermore, miRNAs are frequently dysregulated in various cancer types, acting as tumor suppressor genes or oncogenes^6^. Recently, emerging evidences have shown the dysregulation of miRNAs was closely associated with chemoresistance of different tumors. For example, miR-125b confers paclitaxel resistance through targeting the pro-apoptotic gene Bak1 in breast cancer^7^. In hepatocellular carcinoma, upregulated miR-130a increases cisplatin-resistance and activation of Wnt/β-catenin signaling by targeting tumor suppressor gene RUNX3^8^. In contrast, some miRNAs inhibit drug resistance as tumor suppressor roles. For instance, miR-100 resensitizes docetaxel-resistant lung cancer cells by targeting Plk1^9^. Moreover, miR-199-3p were found to improve the sensitivity to doxorubicin of hepatocarcinoma cells via suppressing mTOR and c-Met^10^. Even more encouraging, miR-34a and miR-122 have entered clinical trials as two miRNA-based drugs for liver cancer patients^11,12^. Therefore, the development of miRNA-based combinational therapy is promising in clinical practice, requiring us to explore the underlying mechanisms of miRNA-mediated chemoresistance or chemosensitivity.

In current study, we focused on miR-93 which was significantly downregulated in chemoresistant cell lines and clinical tumor samples of breast cancer. We found that miR-93 markedly inhibited cell proliferation and cell cycle progression, whereas promoted PTX-induced apoptosis in vitro. MiR-93 also suppressed tumor growth and enhanced the therapeutic effect of PTX in vivo. Mechanistically, we identified that miR-93 directly targeted E2F1 and CCND1, which in turn repressed pRB/E2F1 pathway and AKT phosphorylation. Collectively, our findings shed light on miR-93 as a potential therapeutic target to overcome chemoresistance of breast cancer.

## Materials and methods

### Differential expression analysis of miRNAs

The miRNA microarray data were available with accession number GSE87570 in GEO database (www.ncbi.nlm.nih.gov/geo/). Data were normalized and then conducted the differential expression analysis using R package limma^13^ from the bioconductor project (http://www.bioconductor.org/). Differential-expressed miRNAs (DEmiRNAs) were detected between BCap37 and Bats-72 cell lines. Adjust *P* value <0.05 and |log_2_ fold change (log_2_ FC)|>1 were set as the thresholds for identifying DEmiRNAs. The detailed miRNAs information was showed in Table S1; the red font and the blue font, respectively, represent upregulated (*n* = 20) and downregulated (*n* = 14) DEmiRNAs. The top 10 up-/downregulated DEmiRNAs were sorted by fold change, and were depicted the heatmap using R package pheatmap from the CRAN project (https://cran.r-project.org/).

### Human breast cancer cell lines

The human breast cancer cell line BCap37 were purchased from the Cell Bank of the Chinese Scientific Academy. The chemoresistant cell lines, Bats-72 and Bads-200, were established by PTX treatment of parental BCap37 cells^14^. All cells were cultured in Roswell Park Memorial Institute (RPMI) 1640 medium (31800105, Gibco, Life Technologies, Carlsbad, CA, USA) with 10% fetal bovine serum (FBS; 04-0101-1, Biological Industries, Cromwell, CT, USA), and were incubated at 37 °C with 5% CO_2_ in a water-jacketed incubator (Thermo Scientific, Waltham, MA, USA). The cell culture medium was changed every 2 days.

### Clinical breast cancer specimens

The frozen breast cancer tissues and adjacent normal tissues were collected from Zhejiang Cancer Hospital of the University of Chinese Academy of Sciences (*n* = 49). Our study was approved by the Ethical Committee of Zhejiang Cancer Hospital, and informed consent was obtained from each patient prior to surgery. The clinical data of tumor samples were listed in Table S2.

### Quantitative real-time PCR (qRT-PCR)

Total RNA was extracted from cell lines or frozen tissues using RNAiso plus Reagent (9109, TaKaRa biotechnology, Kusatsu, Japan), and then was reverse transcribed into complementary DNA (cDNA) using PrimeScript RT Reagent kit (RR037A, TaKaRa biotechnology). The qRT-PCR was performed using Roche LightCycler480 II Real-time PCR Detection System with TB Green^®^ Premix Ex Taq^™^ (Tli RNaseH Plus) (RR420A, TaKaRa Biotechnology). Quantification of miRNAs was performed with stem-loop RT-PCR. All reactions were run in triplicate, and were calculated by the comparative threshold method (2^−ΔΔCt^), miR-93 expression relative to U6 and genes expression relative to GAPDH. The sequences of primers for qRT-PCR were listed in Table S4.

### The validation of miR-93 promoter DNA methylation with TCGA data

The expression data of miR-93, MCM7, E2F1, and CCND1 in breast cancer samples were retrieved from The Cancer Genome Atlas (TCGA) data portal (https://genome-cancer.ucsc.edu/) updated by the end of March 31, 2017. The methylation data of miR-93 gene promoter were analyzed using the gene chip data Illumina Infinium Human DNA Methylation 450 K downloaded from the University of California Santa Cruz Xena Cancer Browser (UCSC) (http://xena.ucsc.edu/). The DNA methylation level at each CpG site on the miR-93 gene promoter was calculated by *β* value (*β* = methylated raw value/(methylated raw value + unmethylated raw value + 100)), ranging from 0 (completely unmethylated sites) to 1 (completely methylated sites). The median *β* value of CpG sites on the miR-93 gene promoter was used to assess the methylation status of each TCGA sample. We compared the methylation levels of miR-93 promoter region in paired tumor and adjacent normal samples (*n* = 90, respectively). Moreover, we validated the correlations between the methylation of miR-93 promoter locus and the expressions of miR-93, MCM7, E2F1, and CCND1 using TCGA samples (*n* = 614), by a linear fitting algorithm in R. The detailed methylation and expression data were listed in Table S3.

### Cell transfection

The mimics/inhibitors of miR-93 (miR-93/in-93) and negative control (NC/in-NC) were purchased from Ribobio (Guangzhou, China). The siRNAs of E2F1 (siE2F1), CCND1 (siCCND1), and negative control (siNC) were purchased from GenePharma (Shanghai, China). The sequence of siE2F1: (sense) 5′-GCCUGGGUGAUUUAUUUAUUU-3′, (antisense) 5′-AUAAAUAAAUCACCCAGGCUU-3′; the sequence of siCCND1: (sense) 5′-CAGGCACGGUUUGGAAAUAUU-3′, (antisense) 5′-UAUUUCCAAACCGUGCCUGUU-3′. Above miRNA mimics, inhibitors, siRNAs, and E2F1/CCND1-overexpressing plasmids were transfected into cells using Lipofectamine^TM^ 3000 according to the manufacturer’s instructions. Twelve hours after transfection, cells were changed fresh cell culture medium for following experiments. The sequences of primers used to construct E2F1/CCND1-overexpressing plasmids were listed in Table S4.

### MTT cell viability assay

Cells were evenly added into 96-well plates with 2.5 × 10^3^ cells per well. Twelve hours later, each column was transfected with specific treatments. Cells were incubated for various durations as indicated after that 15 μl of MTT (5 mg/ml) solution were added into each well. After additional 4 h in the incubator, the absorbance of each well was measured under 570 nm wavelength. Cell growth curves were depicted in Graphpad Prism 7 software. For IC50 of PTX, “relative cell viability” was calculated, i.e., the cell viability of drug-feed wells/the cell viability of drug-free wells, and further fitted to a dose–response curve in Graphpad Prism 7 software.

### Colony formation assay

Cells were evenly plated into 6-well plates (300 cells per well). Twelve hours later, miR-93 and NC mimics were, respectively, transfected into BCap37, Bats-72, and Bads-200 cells. After 12 h of transfection, the cell culture medium was changed every 2 days till the 12th days. Cells were fixed with 4% Paraformaldehyde (MA0192, Dalian Meilun Biotechnology, China) for 20 min. The fixed cells were washed by PBS, and then were dyed with 0.1% Crystal violet staining solution for 30 min. Finally, the number of viable colonies were counted using ImageJ software.

### Flow cytometry analysis

Cells were plated in 12-well plates at 4 × 10^4^ cells per well for flow cytometry analyses of cell cycle and apoptosis. Twelve hours later, cells were treated with specific reagents. After 12 h of transfection, each well was changed cell culture medium with or without PTX for another 72 h of incubation (PTX concentrations: 10 nM for BCap37, 200 nM for Bats-72, 2000 nM for Bads-200). The cell cycle staining kit (MultiSciences Biotech, China) was used to detect cell cycle, and AnnexinV-FITC/PI apoptosis kit (MultiSciences Biotech, China) was used to measure cell apoptosis, according to the manufacturer’s instructions. The flow cytometry analysis was performed in BD FACSCalibur™ flow cytometry system.

### In vivo studies

All animal study procedures were approved by Laboratory Animal Welfare and Ethics Committee of Zhejiang University. The lentivirus vectors of miR-93 and control were purchased (Vigene Biosciences, Shandong, China), and were efficiently delivered into Bads-200 cells to establish a stable miR-93-overexpressing cell line and NC cell line, respectively (Fig. S1c). A total of 3 × 10^6^ cells in 0.2 ml PBS were subcutaneously inoculated into the right flank regions of immune-deficient nude mice (female, 3–6-weeks old, 10 mice, respectively). After 5 days, each group of 10 mice were randomly divided into two subgroups with intraperitoneal injection of PBS or PTX (10 mg/kg) every 5 days for total of five cycles. The maximum (*L*) and minimum (*W*) length of the tumor were measured every 5 days using a slide caliper, and the tumor volume was calculated by the formula *V* = 1/2(*L* × *W*^2^). The curves of tumor growth were depicted based on tumor volume and corresponding time (days) after treatment. Finally, the tumor tissues were removed and weighted after the animals were terminated.

### Immunohistochemistry (IHC) staining

Antibodies specific against E2F1 (ab179455, Abcam), CCND1 (ab134175, Abcam), and Ki67 (ab15580, Abcam) were used for IHC staining of 4-μm-thick paraffin-embedded sections of xenograft tumor samples. The staining was visualized using the DAKO Envision kit (DAKO, CA). Slides were photographed using an optical microscope (Olympus). The quantification of Ki67 were assessed using ImageJ software.

### Dual-luciferase reporter assay

The 3′-UTRs of E2F1/CCND1 were amplified and inserted into psiCHECK-2 (Promega) vector to construct wide-type (wt) vectors. Both of the inserted regions contain two putative target sites with miR-93 (Fig. S1h, i). Then the binding sites were mutated to mutant type (mut) vectors, that is mut1, mut2, and mut1 + 2 (Fig. S1h, i). For dual-luciferase reporter assay, Bads-200 cells were evenly distributed into a 96-well plate with 1 × 10^4^ cells per well. Twelve hours later, specific miRNA mimics and wt/mut-reporter vectors were co-transfected for another 24 h incubation. The luciferase activity was measured by the Reporter Assay System Kit (017319, Promega), and “Relative luciferase activity” was calculated, i.e., Firefly luciferase activity/Renilla luciferase activity. The sequences of primers used to construct wt/mut reporter plasmids were listed in Table S4.

### Western blotting

The proteins were extracted from cells, and were measured concentrations using a BCA protein assay kit (Beyotime Biotec, China). Protein samples were fractionated using 4–20% SDS-PAGE gels and then transferred to PVDF membranes (Millipore, NY, USA). After 1 h blocking with 5% non-fat milk at room temperature, the membranes were then incubated for 12 h at 4 °C with rabbit anti-human primary antibodies: E2F1 (#3742, CST), RB (#9313, CST), pRB (#8516, CST), PCNA (#13110, CST), c-myc (#5605, CST), pAKT (#4060, CST), CCND1 (ab134175, Abcam), CCNE1 (ab33911, Abcam), CCNA2 (ab181591, Abcam), MDR1 (ab170904, Abcam), MRP1 (ab233383, Abcam), BCRP (ab207732, Abcam), AKT (db1607, Diagbio), Bcl-2 (db2374, Diagbio), Bcl-xl (db225, Diagbio), Bax (db819, Diagbio), GAPDH (db106, Diagbio), and Tubulin (AC015, ABclonal) were used as endogenous controls. The proteins were detected by ECL detection solution (Thermo Scientific™) and analyzed by Image Lab software (Bio-Rad).

### Statistical analysis

All experiments were performed at least three times and the data were shown as mean ± standard deviation (SD). Statistical analyses were performed with GraphPad Prism 7 Software. The two-tailed Student’s *t*-test was used to evaluate the difference between two groups of data. Bars indicate the mean ± SD of three independent replicates. *P < 0.05, **P < 0.01, ***P < 0.001, ****P < 0.0001. *P* value < 0.05 was considered statistically significant.

## Results

### MiR-93 is downregulated in chemoresistant breast cancer cells, and its promoter DNA methylation is negatively correlated with the expression of host gene MCM7

**Fig. 1:**
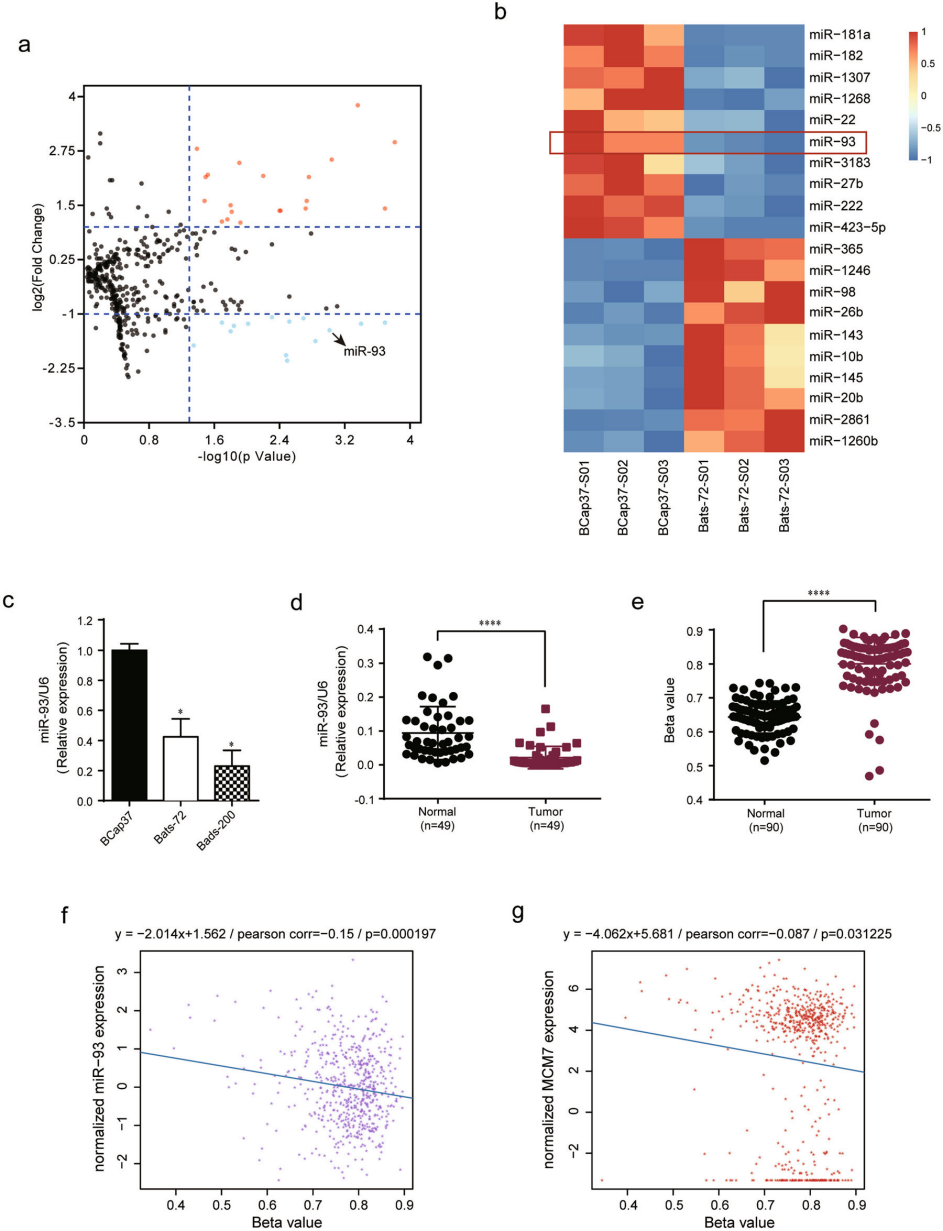
Downregulation of miR-93 is caused by hypermethylation of its host gene MCM7, resulting in chemoresistance of breast cancer. **a** The volcano plot of DEmiRNAs between parental (BCap37) and chemoresistant (Bats-72) breast cancer cell lines. The red dots and blue dots, respectively, represent upregulated (FC > 2, *n* = 20) and downregulated (FC < 0.05, *n* = 14) DEmiRNAs with statistical significance. **b** The heatmap of top 10 up-/downregulated DEmiRNAs (red strips—high expression, blue strips—low expression, columns—samples, rows—miRNAs). **c** The expression of miR-93 expression was measured in two established chemoresistant cell lines (Bats-72, Bads-200), compared with the parental BCap37 cell line. **d** The levels of miR-93 was compared between clinical tumor tissues and their matched adjacent normal tissues (*n* = 49, respectively). **e** The methylation levels of miR-93 promoter region were compared in tumor samples with paired normal samples (*n* = 90, respectively) from TCGA database. **f**, **g** The expression levels of miR-93 and its host gene MCM7 were negatively correlated with the promoter methylation rates of miR-93 in breast cancer samples (*n* = 614) from TCGA database. Bars indicate the mean ± SD of three independent replicates. *P < 0.05, ****P < 0.0001.

Our previous study succeeded in establishing chemoresistant breast cancer cell lines, Bats-72 and Bads-200, by long-term screening with PTX^14^. In addition, we have compared the miRNA expression between chemoresistant cell lines and the parental cell line BCap37 by miRNA microarray^15^. In current study, we focused on miR-93 whose expression was significantly decreased in Bats-72 compared with BCap37 (Fig. 1a, b). Considering the limited accuracy of miRNA microarray, we further measured the expression of miR-93 in BCap37, Bats-72, and Bads-200 cell lines. The result showed that miR-93 was downregulated in two chemoresistant cell lines, especially in Bads-200 (Fig. 1c). Moreover, we detected miR-93 levels in paired clinical specimens (*n* = 49), and found that tumor tissues exhibited lower miR-93 expression (Fig. 1d). Given the promoter DNA methylation is one of mechanisms that cause miRNA downregulation, we assessed the methylation level at miR-93 promoter region using paired breast cancer samples (*n* = 90) in TCGA database. The result showed remarkable hypermethylation of miR-93 promoter locus in tumor samples (Fig. 1e). In addition, by assessing breast cancer tumor samples (*n* = 614) in TCGA database, the promoter methylation rates of miR-93 were found negatively correlated with the expression of miR-93 and its host gene MCM7 (Fig. 1f, g). Overall, miR-93 was downregulated in chemoresistant breast cancer cell lines, that might be caused by the hypermethylation of miR-93 promoter region.

### MiR-93 inhibits cell proliferation, induces G1/S cell cycle arrest and enhances PTX-induced apoptosis of breast cancer cells

**Fig. 2:**
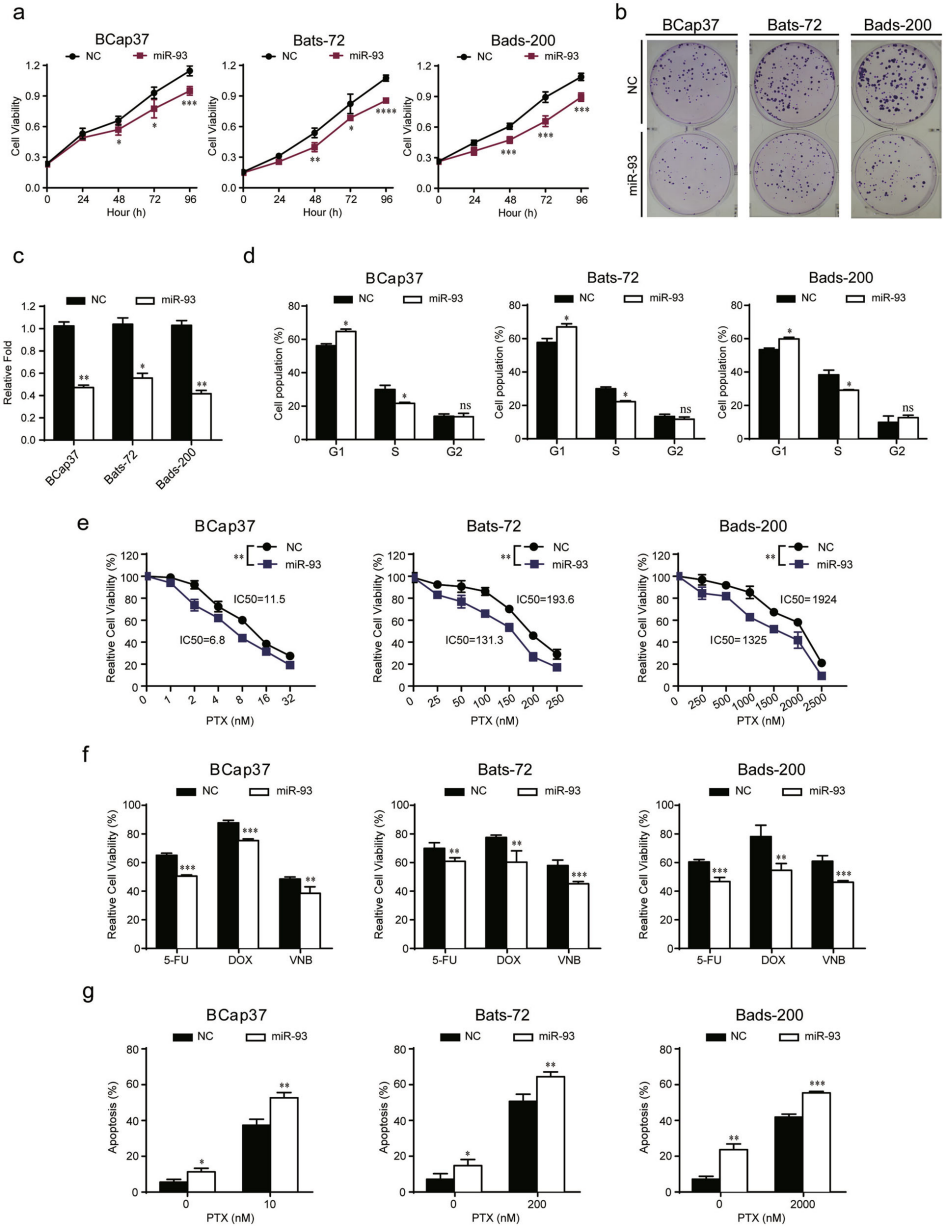
Overexpression of miR-93 attenuates chemoresistance of breast cancer cells via suppressing cell proliferation, inducing G1/S cell cycle arrest, and promoting PTX-induced apoptosis. **a** The cell growth curves were depicted for BCap37, Bats-72, and Bads-200 cells transfected with mimics of NC (NC) or miR-93-5p (miR-93). **b**, **c** The colony formation assay was performed in three cell lines with transfection of NC/miR-93. **d** MiR-93 increased the percentage of G1 phase and reduced the percentage of S phase. **e** The IC50s of PTX in three cell lines were calculated with transfection of NC/miR-93. **f** The sensitivity to other first-line anticancer drugs was increased by miR-93 overexpression (BCap37: 5-FU 15 μM, DOX 200 nM, VNB 20 nM; Bats-72: 5-FU 20 μM, DOX 1.5 μM, VNB 200 nM; Bads-200: 5-FU 20 μM, DOX 13 μM, VNB 3 μM). **g** The apoptosis was evaluated by transfecting NC/miR-93, with or without combinational treatment of PTX. Bars indicate the mean ± SD of three independent replicates. *P < 0.05, **P < 0.01, ***P < 0.001.

In order to explore the correlation between miR-93 and chemoresistance, we evaluated the function of miR-93 on cell proliferation, cycle and apoptosis. The cell viability assays showed that overexpression of miR-93 notably inhibited cell proliferation of BCap37, Bats-72, and Bads-200 (Fig. 2a, Fig. S1a), whereas knockdown of miR-93 promoted the proliferation of 3 cell lines (Fig. S1b, S2a). The colony formation assays further confirmed that miR-93 reduced the clonogenicity of breast cancer cells (Fig. 2b, c). Moreover, miR-93 was found to induce G1/S cell cycle arrest with increased percentage of G1 phase and decreased percentage of S phase (Fig. 2d, Fig. S3a), but knockdown of miR-93 moderately facilitated G1- to S-phase transition (Fig. S2c, e). Next, we assessed whether miR-93 could promote the therapeutic effects of several anti-cancer drugs. The results showed that miR-93 significantly decreased the IC50 of PTX (Fig. 2e), and meanwhile sensitized breast cancer cells to other first-line anticancer drugs, including Fluorouracil (5-FU), Doxorubicin (DOX), and Vinorelbine (VNB) (Fig. 2f). On the other hand, knockdown of miR-93 increased the IC50 of PTX (Fig. S2b). Furthermore, miR-93 notably promoted the apoptosis of BCap37, Bats-72, and Bads-200 cells, with or without PTX (Fig. 2g, Fig. S3b). In addition, knockdown of miR-93 slightly suppressed cell apoptosis with the treatment of PTX (Fig. S2d, f). Taken together, miR-93 suppressed cell proliferation, induced cell cycle arrest and promoted apoptosis, thereby strongly impairing chemoresistance of breast cancer cells.

### MiR-93 inhibits tumorigenesis and improves the therapeutic effect of PTX in vivo

**Fig. 3:**
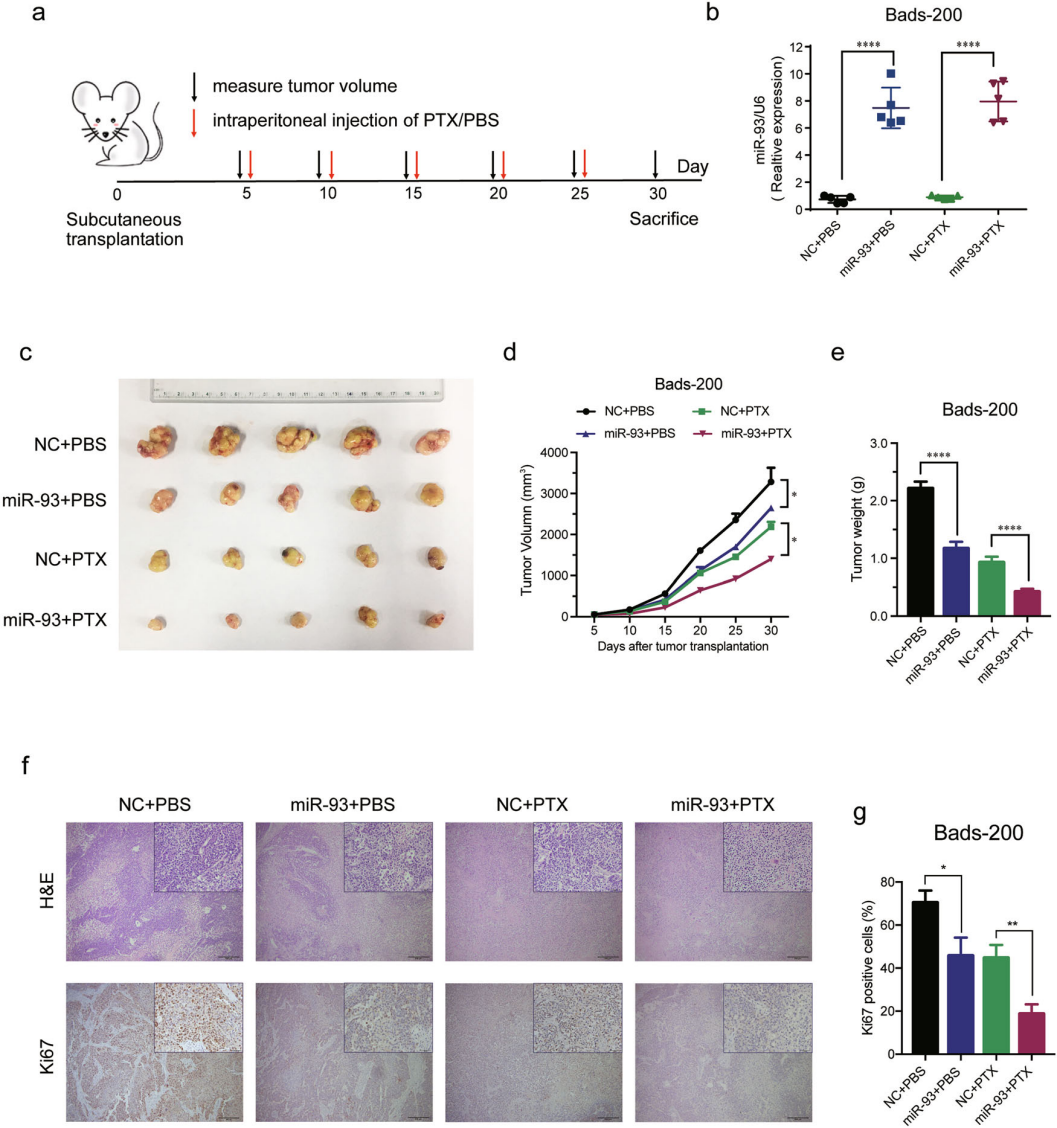
MiR-93 suppresses PTX-resistance and tumorigenesis of breast cancer in vivo. **a** The subcutaneous tumor model was built by Bads-200 cells with stable expression of NC/miR-93, with subsequent treatment of PBS/PTX. **b** The expression levels of miR-93 in transplanted tumor tissues (*n* = 5, respectively) were measured. **c**–**e** MiR-93 significantly reduced tumor volume and weight, especially in the group with combinational treatment of miR-93 and PTX. **f** Representative images of Bads-200 xenografts with H&E or Ki67 staining. Scale bars: (main) 200 μm, (inset) 50 μm. Bars indicate the mean ± SD of three independent replicates. **g** The quantification of Ki67. *P < 0.05, **P < 0.01, ****P < 0.0001.

In view of the lowest miR-93 expression and the strongest PTX-resistant property in Bads-200 cells, they were selected to conduct in vivo study. First, NC/miR-93-overexpressing Bads-200 cells were subcutaneously inoculated into BALB/c nude mice. One week later, the tumor-bearing mice were treated with PTX (10 mg/kg) or PBS every 5 days (Fig. 3a). Actually, the miR-93 expression level of miR-93-overexpressing xenografts increased by ~8 times compared with that of NC (Fig. 3b). Importantly, overexpression of miR-93 significantly reduced tumor volume and weight, and the effect was more pronounced when combined with PTX (Fig. 3c–e). Moreover, Hematoxylin and eosin (H&E) and Ki67 staining implied the cell proliferation was inhibited by overexpression of miR-93 (Fig. 3f, g). Collectively, these results indicated that miR-93 suppressed tumor growth and enhanced the therapeutic effect of PTX in vivo.

### E2F1 and CCND1 are two target genes of miR-93, and they are positively associated with chemoresistance of breast cancer

**Fig. 4:**
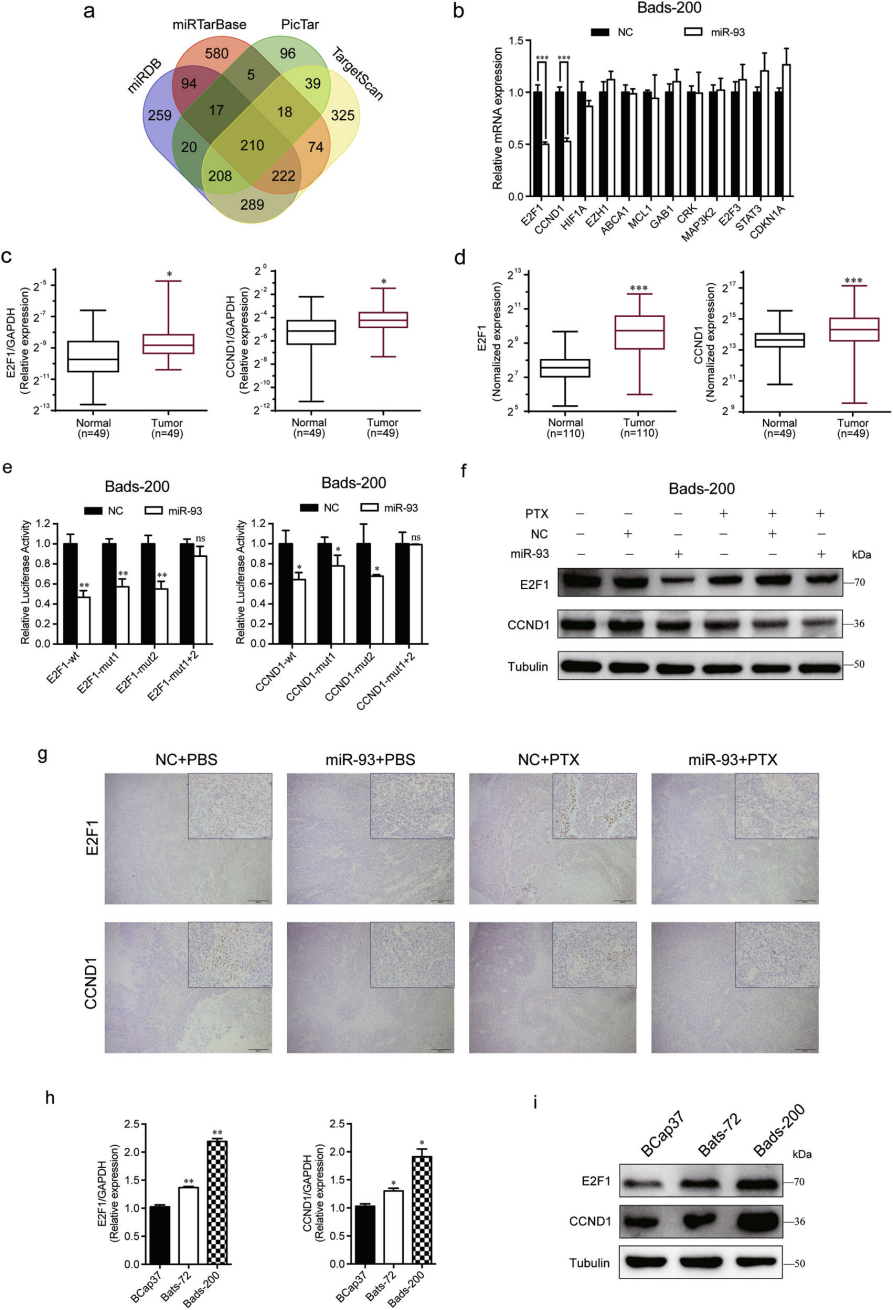
E2F1 and CCND1 are two direct targets of miR-93. **a** 210 targets were predicted for miR-93 in four online databases. **b** The mRNA expression levels of 12 candidate target genes were measured in Bads-200 cells transfected with NC/miR-93. **c**, **d** E2F1 and CCND1 were upregulated in tumor samples of breast cancer, by comparing their mRNA levels in clinical tissues (**c**) and TCGA samples (**d**). **e** The dual luciferase assays were performed in Bads-200 cells. **f** MiR-93 notably downregulated the protein levels of E2F1 and CCND1 in Bads-200 cells, with or without PTX. **g** Representative images of Bads-200 xenografts with IHC staining of E2F1 and CCND1. Scale bars: (main) 200 μm, (inset) 50 μm. **h**, **i** The mRNA and protein levels of E2F1 and CCND1 were measured in BCap37, Bats-72, and Bads-200 cells. Bars indicate the mean ± SD of three independent replicates. *P < 0.05, **P < 0.01, ***P < 0.001.

To investigate the underlying mechanisms of the modulation of miR-93 on chemoresistance, we first studied the expression changes of drug efflux pump and related resistance proteins. It is well known that the ATP-binding cassette (ABC) transporter superfamily can efflux a wide range of chemotherapeutic agents and result in drug resistance^16^, and the prominent members are multidrug resistance protein 1 (MDR1), multidrug resistance-associated protein 1 (MRP1) and breast cancer resistance protein (BCRP). We found that overexpression of miR-93 notably downregulated the mRNA and protein levels of BCRP, but not MDR1 and MRP1 (Fig. S4a, b). Next, we further searched putative target genes of miR-93 in four online miRNA target prediction databases (TargetScan, miRTarBase, PicTar, miRDB). There were 210 target genes included in all four databases (Fig. 4a). The enrichment analyses of Kyoto Encyclopedia of Genes and Genomes (KEGG) and Gene oncology (GO) were performed to better understand the biological significance of 210 targets. The results showed that these target genes were enriched in multiple cancer types and cancer-related pathways (Fig. S5a), and were involved in cell growth, cell cycle G1/S phase transition, transcription factor complex, etc (Fig. S5b). Among 12 candidate target genes, E2F1 and CCND1 were significantly downregulated by miR-93 (Fig. 4b), and they were overexpressed in tumor tissues compared with matched adjacent normal tissues of clinical specimens (Fig. 4c). The analysis of TCGA samples further confirmed the high expression of E2F1 and CCND1 in tumor samples (Fig. 4d).

In order to explore whether miR-93 directly binds to 3′-UTRs of E2F1/CCND1, we performed dual-luciferase assays in Bads-200 cells. The results showed that miR-93 decreased the luciferase activities of wide type and single binding site mutation of E2F1/CCND1, whereas no reduction was observed with double binding sites mutation, proving that miR-93 directly targeted E2F1 and CCND1 with more than one binding sites (Fig. 4e). Furthermore, overexpression of miR-93 greatly attenuated the protein levels of E2F1 and CCND1 in Bads-200 cells (Fig. 4f), consistent with the IHC result in Bads-200-Xenograft mice (Fig. 4g). In addition, the mRNA and protein levels of E2F1 and CCND1 were upregulated in chemoresistant cell lines, implying E2F1 and CCND1 were positively related to chemoresistance (Fig. 4h, i). More intriguingly, we found the expression levels of E2F1 and CCND1 were positively correlated with the methylation of miR-93 promoter locus (Fig. S4c, d), using breast cancer samples (*n* = 614) in TCGA database. In other words, the hypermethylation of miR-93 promoter region not only caused the downregulation of miR-93 (Fig. 1e), but also induced the overexpression of E2F1 and CCND1 (Fig. S4c, d).

Overall, we confirmed E2F1 and CCND1 were two direct targets of miR-93, and proved high expression of E2F1 and CCND1 were positively associated with chemoresistance of breast cancer.

### The inhibitory effects of miR-93 on cell proliferation and cell cycle progression were abrogated by E2F1 and CCND1 via pRB/E2F1 pathway

**Fig. 5:**
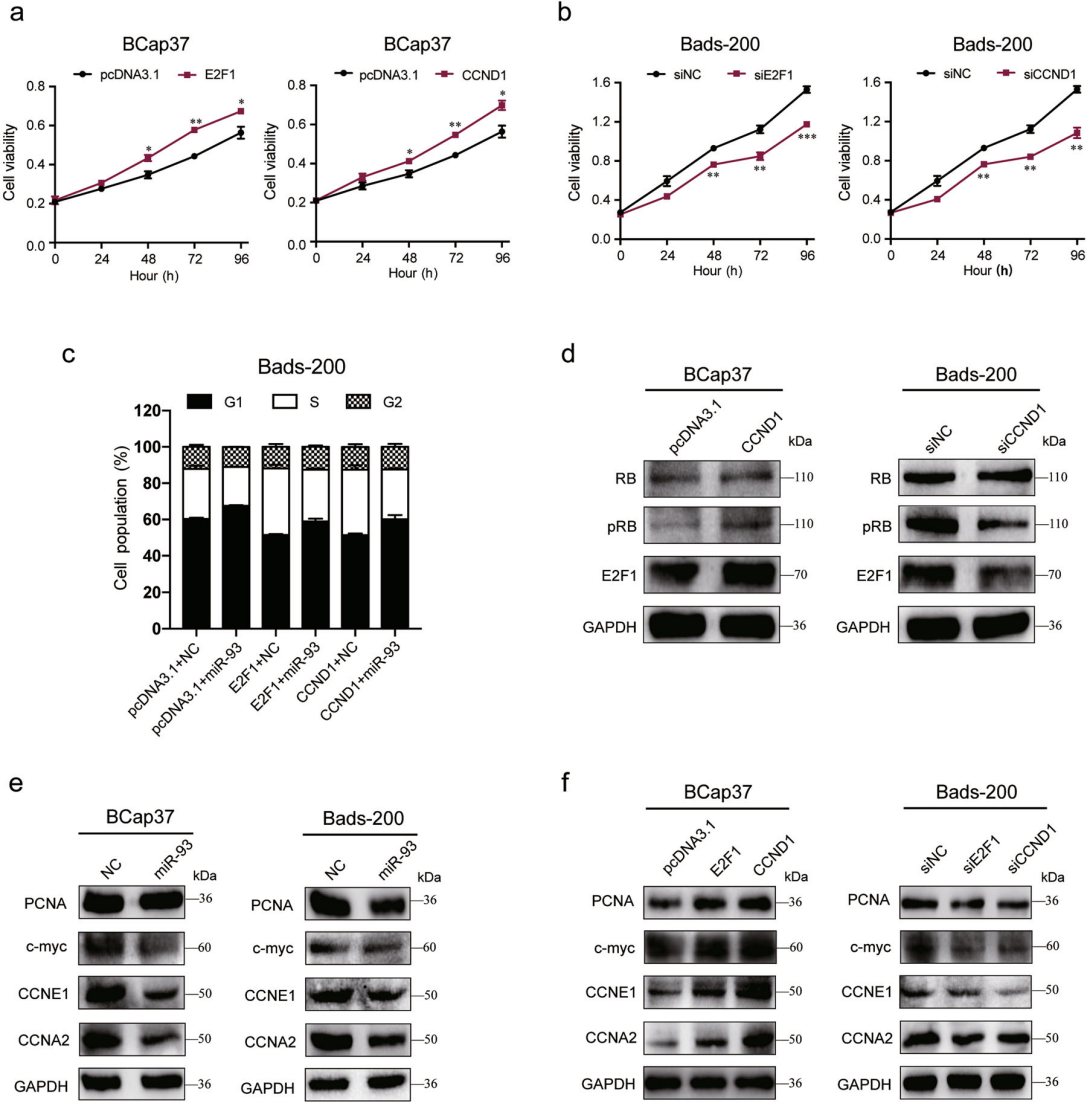
MiR-93 suppresses cell proliferation and cell cycle progression of breast cancer cells via inhibiting E2F1 and CCND1. **a**, **b** The cell growth curves were depicted for BCap37 cells with overexpression of E2F1/CCND1 (**a**), and for Bads-200 cells with knockdown of E2F1/CCND1 (**b**). **c** MiR-93 reversed E2F1/CCND1-induced cell cycle promotion. **d** The pRB/E2F1 activity was increased by overexpression of CCND1 in BCap37 cells, but suppressed by knockdown of CCND1 in Bads-200 cells. **e**, **f** The protein levels of several E2F1 targets were measured in BCap37 and Bads-200 cells with specific treatments. Bars indicate the mean ± SD of three independent replicates. *P < 0.05, **P < 0.01, ***P < 0.001.

Considering the lowest expression of E2F1 and CCND1 in BCap37 cells but their highest level in Bads-200 cells, we, respectively, overexpressed E2F1/CCND1 in BCap37 cells (Fig. S1d, e) whereas knockdown them in Bads-200 cells (Fig. S1f, g). The growth curves showed that overexpression of E2F1/CCND1 increased the cell viability of BCap37 cells, whereas knockdown of E2F1/CCND1 inhibited the cell proliferation of Bads-200 cells (Fig. 5a, b). More importantly, we found miR-93 could reverse E2F1/CCND1-induced promotion of G1/S cell cycle transition (Fig. 5c, Fig. S3c). Given that CCND1 is one of the central components of the G1/S-phase checkpoint regulating the pRB/E2F1 pathway^17^, we tested the regulation of CCND1 on pRB/E2F1 activity in breast cancer cells. The results showed that overexpression of CCND1 in BCap37 cells activated the phosphorylation of RB (pRB) and E2F1, while knockdown of CCND1 in Bads-200 cells antagonized pRB and E2F1 (Fig. 5d). On the other hand, we also evaluated the effect of miR-93 on several E2F1 targets (PCNA, c-myc, CCNE1, CCNA2), and found that overexpression of miR-93 notably suppressed these E2F1 targets (Fig. 5e). Furthermore, knockdown of E2F1/CCND1 in Bads-200 cells also repressed the expression of these E2F1 targets, but opposite trends were observed in BCap37 cells with overexpression of E2F1/CCND1 (Fig. 5f). All these data suggested that the inhibitory role of miR-93 on cell proliferation and G1/S-phase transition was conducted by antagonizing E2F1 and CCND1, subsequently inactivating pRB/E2F1 pathway and several E2F1 downstream targets.

### MiR-93 increases apoptosis by suppressing E2F1/CCND1 and AKT phosphorylation

**Fig. 6:**
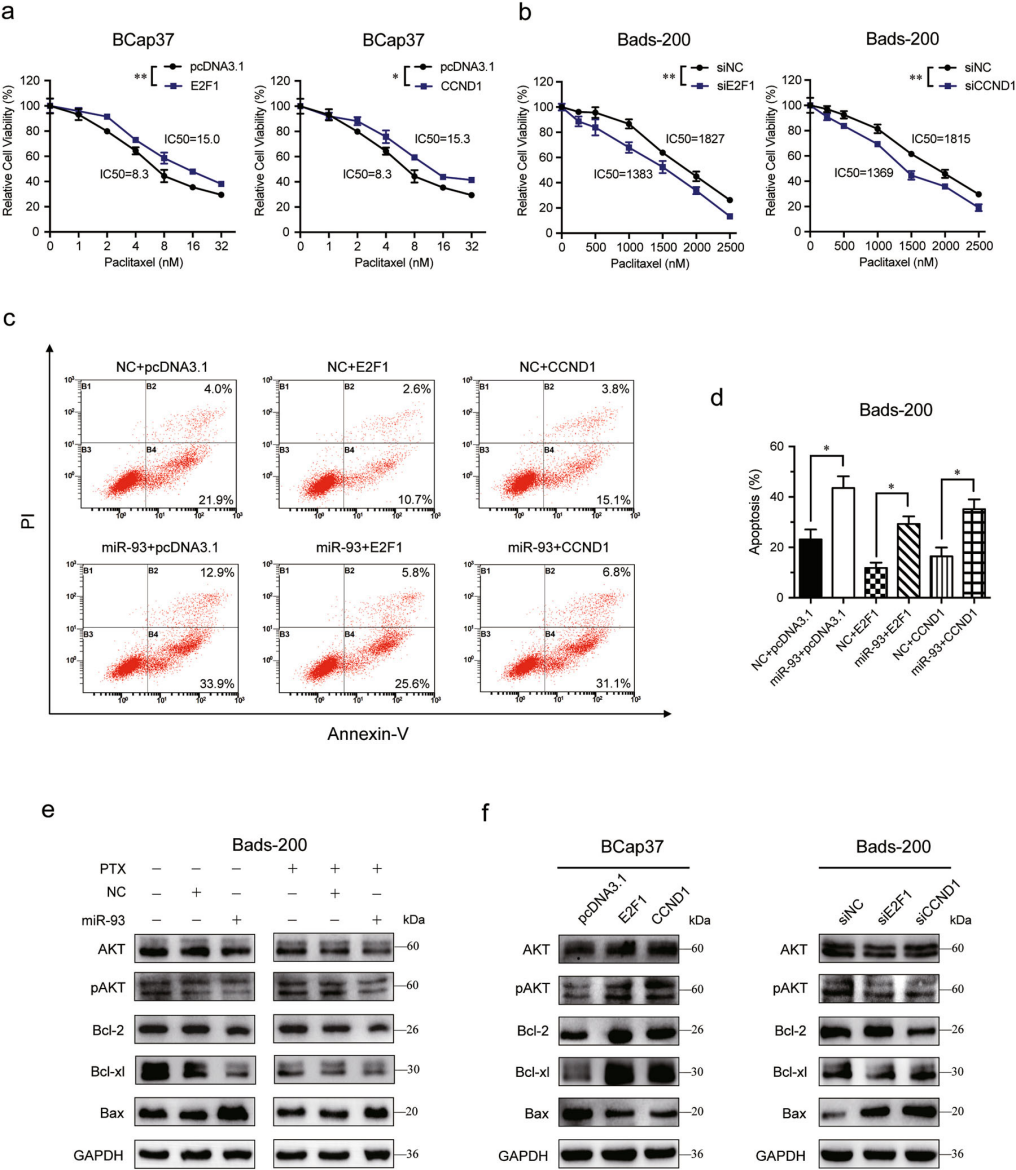
MiR-93 promotes apoptosis via repressing E2F1/CCND1 activity and AKT phosphorylation. **a**, **b** The IC50 of PTX was calculated in BCap37 cells overexpressed with E2F1/CCND1 (**a**), and in Bads-200 cells with E2F1/CCND1 knockdown (**b**). **c**, **d** MiR-93 rescued the E2F1/CCND1-mediated apoptosis reduction in Bads-200 cells. **e**, **f** The protein levels of AKT, pAKT, Bcl-2, Bcl-xl, and Bax were measured in BCap37 and Bads-200 cells according to specific treatments. Bars indicate the mean ± SD of three independent replicates. *P < 0.05, **P < 0.01.

To elucidate whether PTX-sensitivity and miR-93-induced increase of apoptosis were mediated by repression of E2F1 and CCND1, gain-of-function studies were performed in breast cancer cells. The cell viability assays showed that overexpression of E2F1/CCND1 increased the IC50 of PTX in BCap37 cells, whereas knockdown of E2F1/CCND1 improve the sensitivity to PTX in Bads-200 cells (Fig. 6a, b). Furthermore, overexpression of E2F1/CCND1 reduced the apoptosis of Bads-200 cells, and miR-93 could restore E2F1/CCND1-induced apoptotic suppression (Fig. 6c, d). Moreover, miR-93 was found to inhibit the phosphorylation of AKT (pAKT) and anti-apoptotic proteins (Bcl-2, Bcl-xl), but increase the level of pro-apoptotic protein (Bax) in Bads-200 cells (Fig. 6e). In addition, overexpression of E2F1/CCND1 in BCap37 cells increased pAKT, Bcl-2, Bcl-xl and decreased Bax, whereas knockdown of E2F1/CCND1 in Bads-200 cells were observed inverse tendencies (Fig. 6f). Collectively, miR-93 promotes apoptosis of breast cancer cells via repressing E2F1 and CCND1, thereby mediating pAKT activity and apoptosis-related proteins.

## Discussion

Worldwide, breast cancer is the most common malignancies in women, greatly impairing female health. Chemotherapy is one of the primary therapeutic strategies for breast cancer. However, chemoresistance remains a major problem that leads to treatment failure or tumor recurrence. Therefore, it is urgent to develop effective therapies to overcome chemoresistance of breast cancer.

Some upfront studies have demonstrated the regulation of miRNAs on chemosensitivity, potentially served as ideal diagnostic and therapeutic targets^18,19^. In our study, we found that miR-93 was significantly downregulated in chemoresistant breast cancer cell lines and in tumor tissues of clinical breast cancer samples. By analyzing breast cancer patient data retrieved from TCGA database, hypermethylation at miR-93 promoter locus was observed in tumor samples, that could explain the downregulation of miR-93 in chemoresistant cell lines and clinical tumor tissues. Previous studies have demonstrated that miR-93 inhibits invasive and EMT of breast cancer cells^20,21^. Moreover, Liu et al. have proved that miR-93 could modulate the states and fates of breast cancer stem cells^22^. But controversially, miR-93 has also been reported to play an oncogenic role during the occurrence and development of breast cancer^23,24^. These paradoxical findings indicated that the explicit function of miR-93 should be textualized regarding to specific cell types and treatments. To date, very few studies have focused on the regulation of miR-93 on chemosensitivity in breast cancer^25,26^, and to the best of our knowledge, the definitive effect of miR-93 on the chemosensitivity of PTX has not been reported. Nevertheless, given that miR-93 belongs to miR-106b-25 cluster which shares a high degree of homology with the miR-17-92 cluster, miRNAs of these two clusters might exhibit accordant biological functions. For example, miR-17-5p was found to inhibit the cell proliferation^27^, and miR-20a-5p could improve the therapeutic effect of multiple anticancer drugs including PTX^15^. Our present study validated for the first time that miR-93 has broad functions in vitro and in vivo, including suppression of cell proliferation and cell cycle progression, as well as promotion of apoptosis and chemosensitivity of breast cancer.

Remarkably, E2F1 and CCND1 were identified as two direct targets of miR-93 and reversed miR-93-mediated suppression of cell growth and PTX-resistance. E2F1 is known as the best-studied transcription factor of E2F family, controlling cell cycle progression and cell death^28^. CCND1, also termed Cyclin D1, is a well-established oncogene that is commonly amplified in human cancers^29^. Evidences have shown that the amplification of Cyclin D1 promotes the phosphorylation of RB (pRB), subsequently triggering E2F1 release and activating gene transcription^30^. Consistently, our current study found that the protein levels of pRB and E2F1 were increased by overexpression of CCND1 in BCap37 cells, whereas decreased by knockdown of CCND1 in Bads-200 cells. We also proved that miR-93 antagonized several E2F1 downstream targets related to cell cycle progression. Recently, emerging evidences have shown that E2F1 and CCND1 are involved in chemoresistance of various cancers. For instance, high expression of E2F1 induces oxaliplatin-resistance in colorectal cancer^31^. Moreover, CCND1 has been found to increase cisplatin resistance in pancreatic cancer^32^ and 5-fluorouracil resistance in gastric cancer^33^. However, the explicit effects of E2F1 and CCND1 on chemoresistance of breast cancer remains poorly understood. Our findings uncovered for the first time that E2F1 and CCND1 positively modulated PTX-resistance in breast cancer cells. We also showed that miR-93-induced apoptosis was reduced by inhibiting E2F1/CCND1-mediated AKT phosphorylation and thereby restored cell sensitivity to PTX. Previous studies have revealed that E2F1 and CCND1 confer survival advantages through the activation of AKT pathway^34–36^. Indeed, AKT suppressed cell apoptosis by phosphorylating and inhibiting various proapoptotic effectors^37^, playing a vital role in carcinogenesis and chemoresistance^38^. Collectively, we hold the opinion that miR-93 exhibited a tumor suppressor role by targeting E2F1 and CCND1, thereby inactivation of pRB/E2F1 pathway and AKT phosphorylation.

**Fig. 7:**
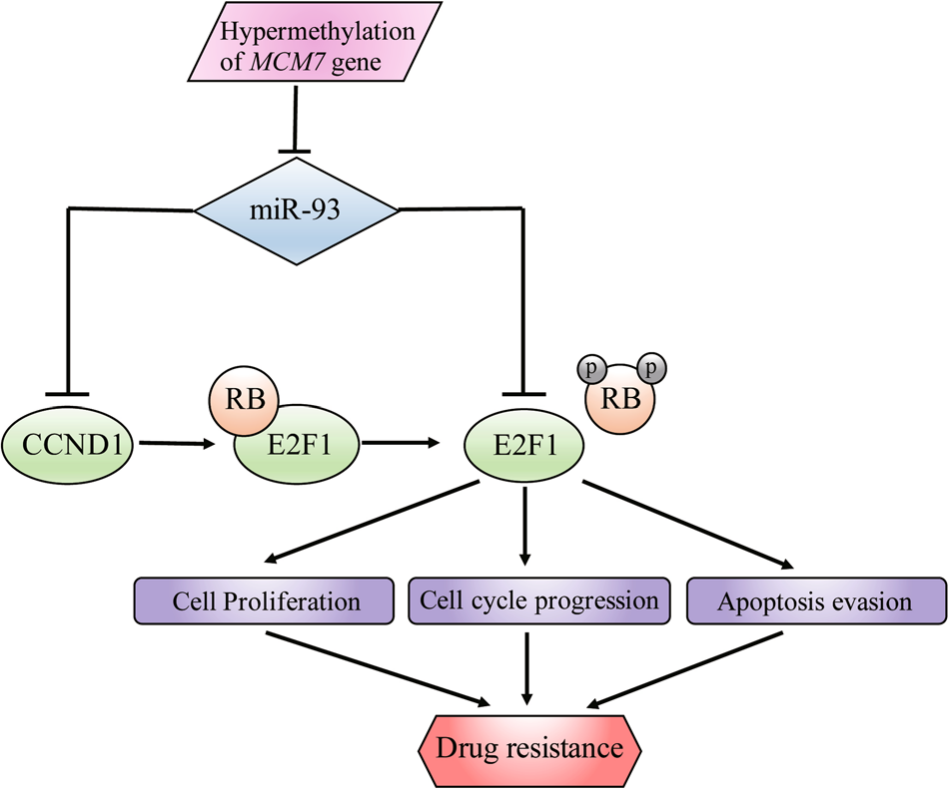
Working model for the regulation of drug resistance by miR-93. Schematic model of miR-93 as a proposed role in regulating drug resistance of breast cancer cells.

Furthermore, considering the heterogeneity and complexity of chemoresistance mechanisms, most efforts that focus on single target or pathway are unable to produce a sustained treatment response. For example, knockdown of cyclin D1 alone are not necessarily sufficient to induce cell death in mantle cell lymphoma (MCL)^39^. Therefore, our study focused on dual targets, and constructed a triplex model consist of miR-93, E2F1, and CCND1. As the model depicted in Fig. 7, the blockade of E2F1 was more efficient owing to the double-inactivation from miR-93 and miR-93-induced suppression of CCND1, resulting in stronger inhibition of E2F1 downstream oncogenes.

In conclusion, we identified that miR-93 downregulation was correlated with cell proliferation and chemoresistance of breast cancer. The mechanistic investigation revealed that miR-93 directly targeted E2F1 and CCND1, thereby suppressing cell cycle progression and apoptosis evasion. Taken together, our study provided a rationale for the treatment with miR-93 to overcome chemoresistance of breast cancer.

## Supplementary information

Supplementary Figure Legends

Figure S1

Figure S2

Figure S3

Figure S4

Figure S5

Table S1

Table S2

Table S3

Table S4
