# Supplementary material for: MiR-93 suppresses tumorigenesis and enhances chemosensitivity of breast cancer via dual targeting E2F1 and CCND1
Source: Cell Death Dis. 2020 Aug 14;11(8):618. doi: 10.1038/s41419-020-02855-6 (PMC7428045; doi:10.1038/s41419-020-02855-6)
Supplement: Supplementary file 1 — Supplementary Figure Legends [file 41419_2020_2855_MOESM1_ESM.docx]

**Supplementary Figure Legends**

**Figure S1**

**a, b** BCap37, Bats-72 and Bads-200 cells were transfected with 50 nM of NC mimics (NC) / miR-93-5p mimics (miR-93), or 100nM of NC inhibitors (in-NC) / miR-93 inhibitors (in-93). Then the expression of miR-93 was measured by qRT-PCR. **c** The efficiency of stable miR-93-expressing Bads-200 cells were measured by qRT-PCR. **d-g** The mRNA and protein levels of E2F1 and CCND1 were measured with specific reagents. BCap37 cells were transfected with negative plasmids (pcDNA3.1) or E2F1/CCND1-overexpressing plasmids (E2F1/CCND1) (**d, e**). Bads-200 cells were transfected with NC siRNAs (siNC), E2F1 siRNAs (siE2F1) or CCND1 siRNAs (siCCND1) (**f, g**). **h, i** The binding sites (blue font) between miR-93 and 3’-UTRs of E2F1/CCND1 in wide type (wt) reporter plasmids. The mutant type (mut) vectors were produced by 6 or 7 bp mutation (red font) at each binding site.

**Figure S2**

**a** The cell growth curves of BCap37, Bats-72 and Bads-200 cells with transfection of inhibitors of NC (in-NC) / miR-93 (in-93). **b** The IC50s of PTX in 3 cell lines were measured with transfection of in-NC/in-93. **c, e** In-93 reduced the percentage of G1 phase and increased the percentage of S phase in BCap37 and Bads-200 cells. **d, f** The apoptosis of BCap37 and Bads-200 with combinational treatment of in-NC/in-93 and PTX were measured. Bars indicate the mean ± SD of three independent replicates. * P < 0.05, ** P < 0.01.

**Figure S3**

**a** The cell cycle of BCap37, Bats-72 and Bads-200 cells transfected with NC/miR-93. **b** The apoptosis was detected in 3 cell lines according to specific treatments. **c** The cell cycle of Bads-200 cells with co-transfection of specific plasmids and siRNAs.

**Figure S4**

**a, b** The mRNA and protein levels of MDR1, MRP1 and BCRP were measured in 3 cell lines with transfection of NC/miR-93. **c, d** The expression levels of E2F1 (**c**) and CCND1 (**d**) were positively correlated with the promoter methylation rates of miR-93 in breast cancer samples (n=614) from TCGA database.

**Figure S5**

**a, b** KEGG enrichment analysis and GO items of target genes of miR-93. GO items includes biological process (BP), cellular component (CC) and molecular function (MF).
