# Supplementary figures and images for: MiR-93 suppresses tumorigenesis and enhances chemosensitivity of breast cancer via dual targeting E2F1 and CCND1
Source: Cell Death Dis. 2020 Aug 14;11(8):618. doi: 10.1038/s41419-020-02855-6 (PMC7428045; doi:10.1038/s41419-020-02855-6)

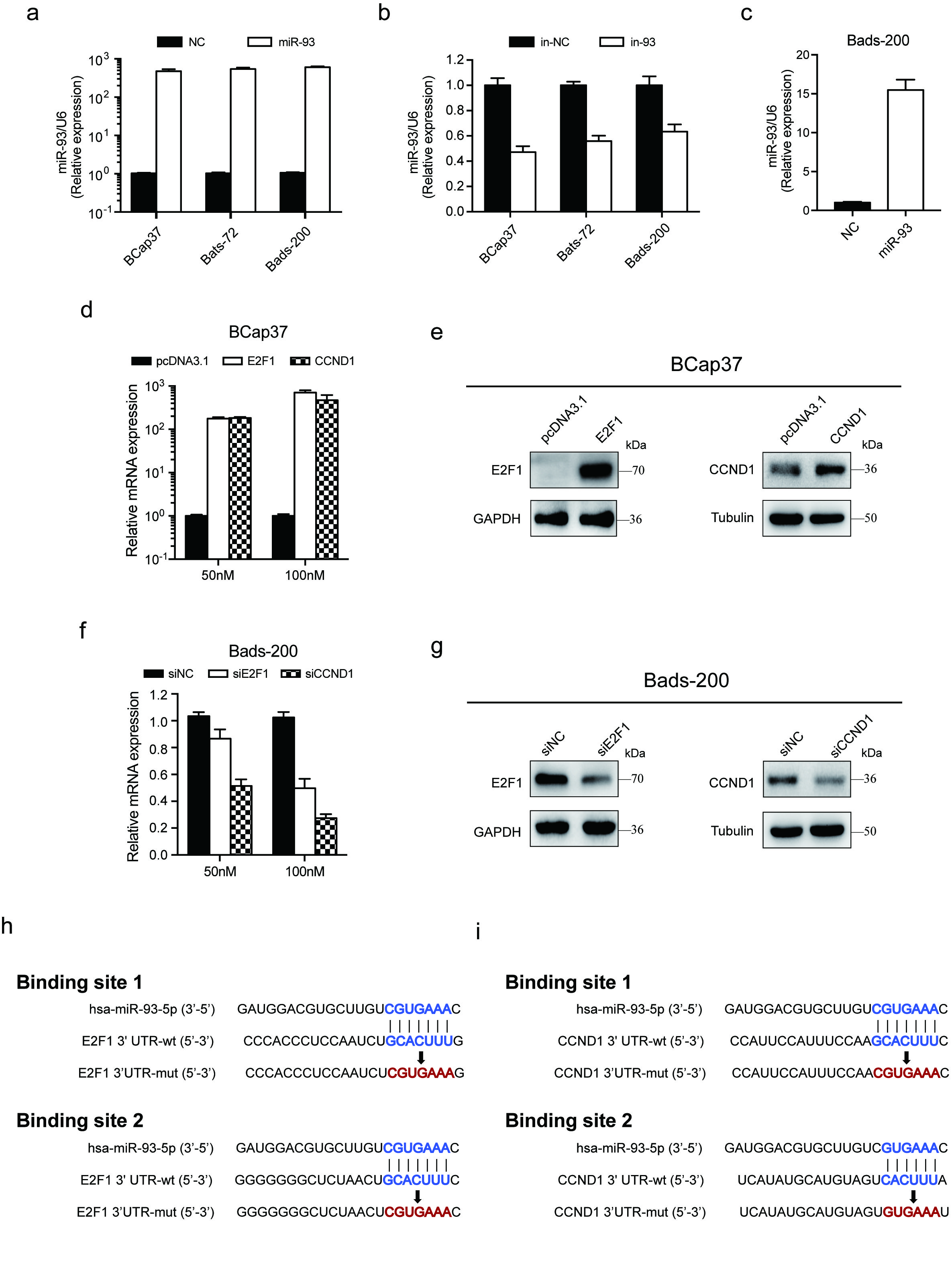

Supplement: Supplementary file 2 — Figure S1 [file 41419_2020_2855_MOESM2_ESM.jpg]

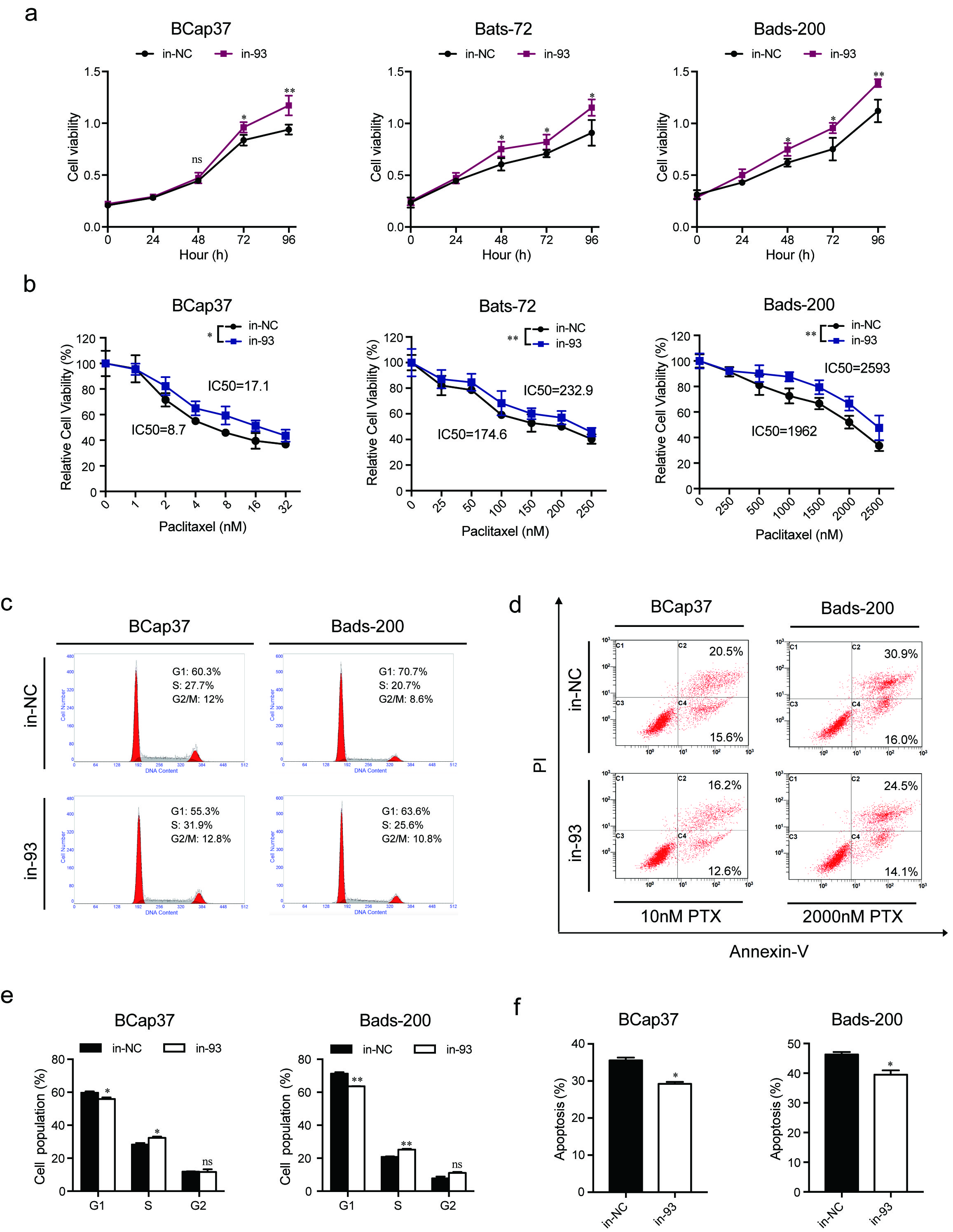

Supplement: Supplementary file 3 — Figure S2 [file 41419_2020_2855_MOESM3_ESM.jpg]

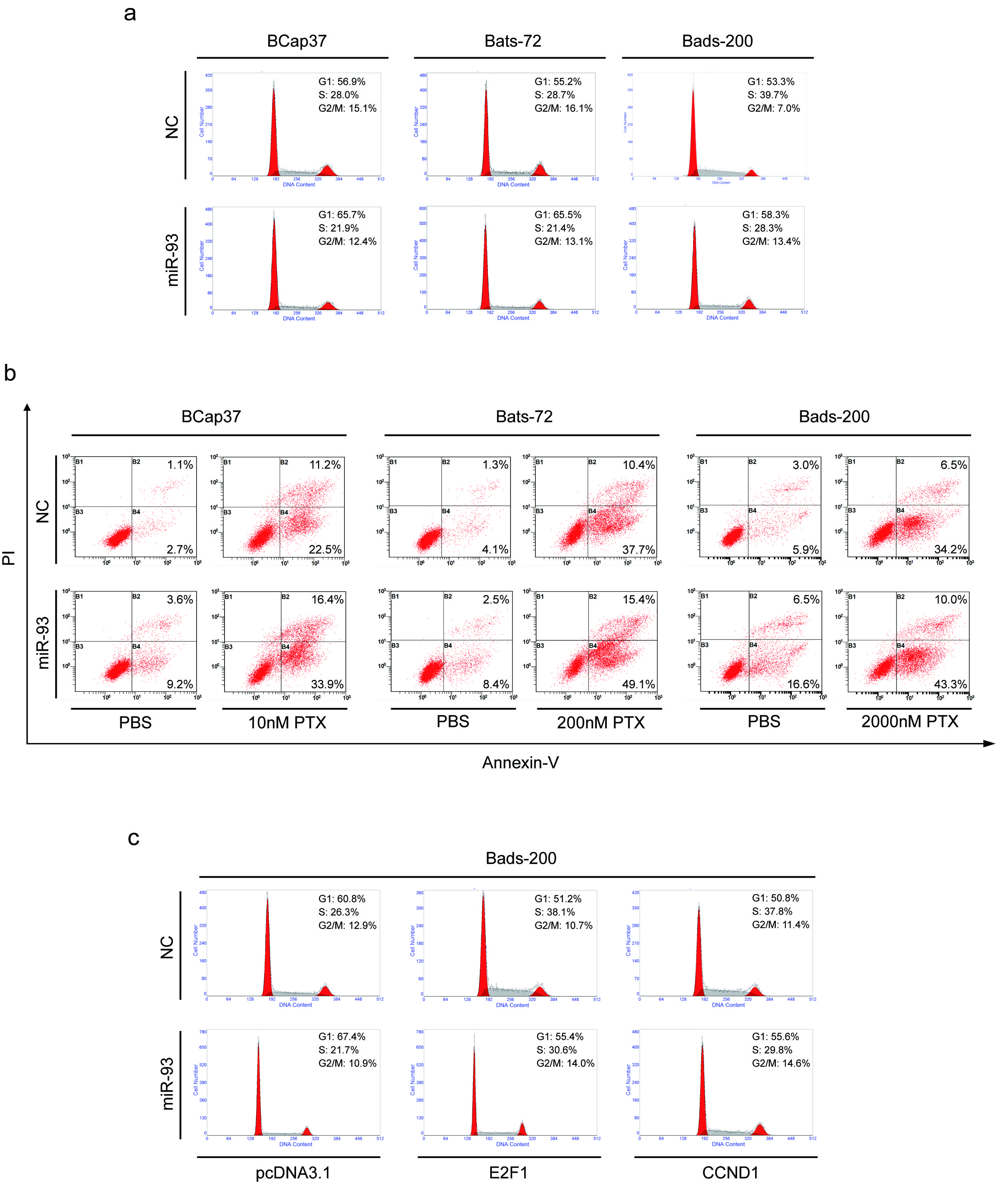

Supplement: Supplementary file 4 — Figure S3 [file 41419_2020_2855_MOESM4_ESM.jpg]

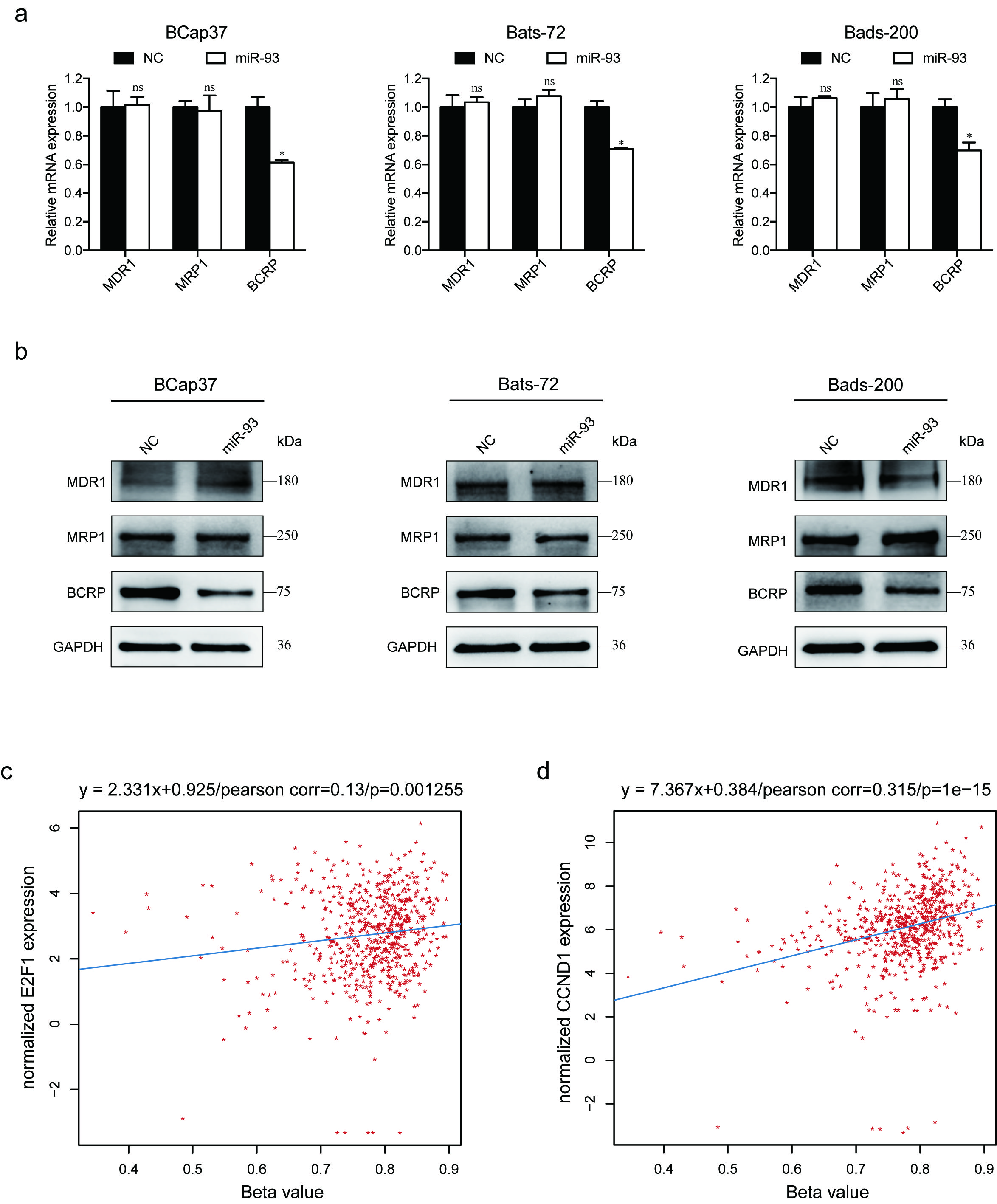

Supplement: Supplementary file 5 — Figure S4 [file 41419_2020_2855_MOESM5_ESM.jpg]

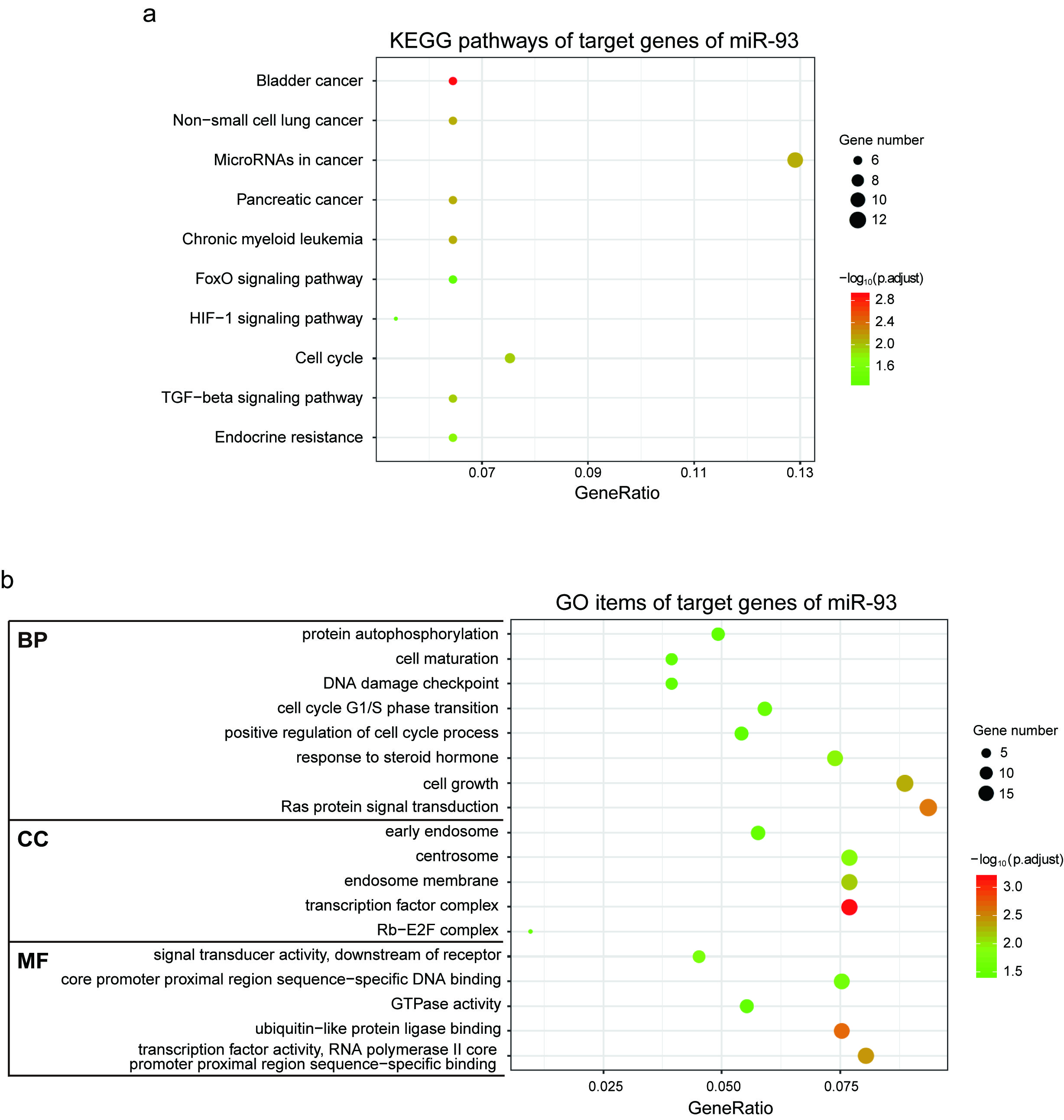

Supplement: Supplementary file 6 — Figure S5 [file 41419_2020_2855_MOESM6_ESM.jpg]
