# Supplementary material for: MiR-93 suppresses tumorigenesis and enhances chemosensitivity of breast cancer via dual targeting E2F1 and CCND1
Source: Cell Death Dis. 2020 Aug 14;11(8):618. doi: 10.1038/s41419-020-02855-6 (PMC7428045; doi:10.1038/s41419-020-02855-6)
Supplement: Supplementary file 8 — Table S2 [file 41419_2020_2855_MOESM8_ESM.docx]

Table S2 The information of clinical samples

| **No.** | **Age at operation (years)** | **Tumor Size**  **(cm)** | **ER** | **PR** | **HER2** | **TNM**  **Stage** | **Histological Grade** |  |
| --- | --- | --- | --- | --- | --- | --- | --- | --- |
| 1 | 76 | 3.5 | 3+ | 3+ | 2+ | T2N2M0 | III |  |
| 2 | 41 | 5 | - | + | + | T2N1M0 | II |  |
| 3 | 53 | 4 | 3+ | - | 3+ | T2N1M0 | II |  |
| 4 | 56 | 3 | 3+ | - | - | T2N0M0 | II |  |
| 5 | 45 | 4 | - | - | 3+ | T2N1M0 | II |  |
| 6 | 33 | 4.5 | - | - | - | T2N0M0 | II |  |
| 7 | 43 | 3 | - | - | 2+ | T2N2M0 | II |  |
| 8 | 53 | 3 | + | + | + | T2N3M0 | III |  |
| 9 | 34 | 2 | 2+ | - | - | T1N2M0 | III |  |
| 10 | 56 | 3.5 | 3+ | + | + | T2N2M0 | III |  |
| 11 | 65 | 3.5 | 2+ | - | - | T2N3M0 | III |  |
| 12 | 37 | 1.8 | 2+ | 3+ | - | T1N3M0 | III |  |
| 13 | 40 | 2 | - | - | - | T1N3M0 | III |  |
| 14 | 68 | 2 | 3+ | + | 2+ | T1N1M0 | II |  |
| 15 | 49 | 8 | - | - | 3+ | T4N3M0 | III |  |
| 16 | 54 | 2 | - | - | + | T1N2M0 | III |  |
| 17 | 43 | 3 | - | - | 3+ | T2N0M0 | II |  |
| 18 | 40 | 4 | 3+ | 3+ | 2+ | T2N0M0 | II |  |
| 19 | 48 | 5 | 3+ | - | 3+ | T2N1M0 | II |  |
| 20 | 52 | 6.5 | - | - | 3+ | T2N2M0 | III |  |
| 21 | 48 | 3 | 2+ | 2+ | 3+ | T2N1M0 | II |  |
| 22 | 38 | 5 | + | + | 3+ | T2N2M0 | III |  |
| 23 | 38 | 2 | - | - | 3+ | T1N0M0 | II |  |
| 24 | 48 | 2 | + | ++ | 2+ | T1N0M0 | I |  |
| 25 | 45 | 1.6 | - | - | + | T1N1M0 | II |  |
| 26 | 44 | 4 | 2+ | + | - | T2N2M0 | III |  |
| 27 | 50 | 2.2 | 2+ | 3+ | 3+ | T1N0M0 | I |  |
| 28 | 55 | 2.2 | - | - | 2+ | T2N0M0 | II |  |
| **No.** | **Age at operation (years)** | **Tumor Size (cm)** | **ER** | **PR** | **HER2** | **TNM Stage** | **Histological Grade** |  |
| 29 | 62 | 1.2 | 2+ | 2+ | + | T1N2M0 | III |  |
| 30 | | 51 | 2.5 | - | - | 3+ | T2N1M0 | II |
| 31 | 54 | 3.5 | - | - | 3+ | T2N1M0 | II |  |
| 32 | 44 | 7 | 2+ | 2+ | 2+ | T3N2M0 | III |  |
| 33 | 43 | 6 | 3+ | 2+ | - | T3N3M0 | III |  |
| 34 | 46 | 2.5 | - | - | + | T2N1M0 | II |  |
| 35 | 40 | 3.5 | - | - | - | T2N0M0 | II |  |
| 36 | 53 | 6 | 2+ | 3+ | 2+ | T3N0M0 | II |  |
| 37 | 49 | 3 | + | + | 2+ | T2N2M0 | III |  |
| 38 | 53 | 4.3 | - | - | 2+ | T2N1M0 | II |  |
| 39 | 46 | 2.5 | 2+ | 3+ | + | T2N0M0 | II |  |
| 40 | 47 | 3 | 2+ | 3+ | 2+ | T2N1M0 | II |  |
| 41 | 54 | 2 | 3+ | + | 2+ | T1N1M0 | II |  |
| 42 | 55 | 1.6 | 3+ | 2+ | 2+ | T1N1M0 | II |  |
| 43 | 64 | 6.5 | 3+ | 2+ | 2+ | T3N0M0 | II |  |
| 44 | 57 | 3.5 | - | - | 2+ | T2N1M0 | II |  |
| 45 | 33 | 4.5 | 3+ | 3+ | 2+~3+ | T2N2M0 | III |  |
| 46 | 44 | 1.6 | 2+ | 2+ | 2+ | T1N0M0 | I |  |
| 47 | 43 | 5.4 | - | - | 3+ | T3N3M0 | III |  |
| 48 | 41 | 3.5 | +~2+ | - | 3+ | T2N0M0 | II |  |
| 49 | 55 | 2.5 | 3+ | 3+ | 2+ | T2N3M0 | III |  |
