# Supplementary material for: MiR-93 suppresses tumorigenesis and enhances chemosensitivity of breast cancer via dual targeting E2F1 and CCND1
Source: Cell Death Dis. 2020 Aug 14;11(8):618. doi: 10.1038/s41419-020-02855-6 (PMC7428045; doi:10.1038/s41419-020-02855-6)
Supplement: Supplementary file 10 — Table S4 [file 41419_2020_2855_MOESM10_ESM.docx]

Table S4 Sequences for primers used in this study

| **Name** | **Sequence** |
| --- | --- |
| **Primers for qRT-PCR**  MDR1-F  MDR1-R  MRP1-F  MRP1-R  BCRP-F  BCRP-R  E2F1-F  E2F1-R  CCND1-F  CCND1-R  HIF1A-F  HIF1A-R  EZH1-F  EZH1-R  ABCA1-F  ABCA1-R  MCL1-F  MCL1-R  GAB1-F  GAB1-R  CRK-F  CRK-R  MAP3K2-F  MAP3K2-R  E2F3-F  E2F3-R  STAT3-F  STAT3-R  CDKN1A-F  CDKN1A-R  GAPDH-F  GAPDH-R  **Primers for 3’UTR**  E2F1-F  E2F1-R  CCND1-F  CCND1-R  mut1-E2F1-F  mut1-E2F1-R  mut2-E2F1-F  mut2-E2F1-R  mut1-CCND1-F  mut1-CCND1-R  mut2-CCND1-F  mut2-CCND1-R  **Primers for overexpressing plasmids**  E2F1-F  E2F1-R  CCND1-F  CCND1-R | GATTGCTCACCGCCTGTCCAC  CGTGCCATGCTCCTTGACTCTG  GGTGTCTCGTTAGAGCCCAAAGTG  AAGTCGGCGGCGTAATTCTTAGC  GCAGCAGGTCAGAGTGTGGTTTC  ACTGAAGCCATGACAGCCAAGATG  CGGTGTCGTCGACCTGAACT  AGCTGGATGCCCTCAAGGAC  ATGCTGAAGGCGGAGGAGAC  CATCCAGGTGGCGACGATCT  AGTTCCGCAAGCCCTGAAAGC  GCAGTGGTAGTGGTGGCATTAGC  GCAGGCAGCACAGACTGAACTC  TCTGGCTAAGGAAGGATGGCGTAG  GCTCAGTGGGATGGATGGCAAAG  CTCCGTCTGGCAATTAGCAGTCTC  GCCGCTGACGCCATCATGTC  CAACTCGTCCTCCTCCTCCTCTG  TGGTGGCTGGCTACTCGGATAC  GACCACTTCACCACCGCTCATG  GGCAGGAGGAGGCGGAGTATG  CACCACTGCTCTTCAGGCTTGTC  CTTTGGCTTGGCTTTGACTTCTGC  ACAGCTCTGGAAGGTGAGTATGGG  GGTCTGCTCACCAAGAAGTTCA  CTGCCTTGTTCAAATCCAATACC  GAGGCAGGAGAATCGCTTGAACC  TCTCAGACTGTCGCCCAGGATG  CAGTTCCTTGTGGAGCCGGA  TTCTGACGGACATCCCCAGC  TCTCCTCTGACTTCAACAGCGAC  CCCTGTTGCTGTAGCCAAATTC  GGCGATCGCTCGAGGGGAATGAAGGTGAACA  TTTATTGCGGCCAGCGGCCGCCTGGATCTGC  TCTAGGCGATCGCTCGAGGAATAGGCATTAA  TTTATTGCGGCCAGCGGCCGCTGGCAATGTGAGAAT  TAACTCGTGAAACGGCCCTTTTGCTCTGGGGG  GGCCGTTTCACGAGTTAGAGCCCCCCCACGCG  CTCCAATCTCGTGAAAGATTTGCTTCCTAACAGCTCTGTTC  CTTTCACGAGATTGGAGGGTGGGGCAGAGCAG  AACGTTGTAGTGTGAAAATAAGTCATTGTATGTTATTATATTCCGTAGG  TTTTCACACTACAACGTTATGAGCAAGCAAAGTACAGAGATGC  TCCAACGTGAAACAGTCCAATAGGTGTAGGAAATAGCG  GACTGTTTCACGTTGGAAATGGAATGGTTTTAGAATAT  AGCACAGTGGCGGCCATGGCCTTGGCCGGGGCC  GCCCTCTAGACTCGATCAGAAATCCAGGGGGGTGAGG  AGCACAGTGGCGGCCATGGAACACCAGCTCCTGTGC  GCCCTCTAGACTCGATCAGATGTCCACGTCCCGC |
